# Prevalence of dementia among Kashmiri migrants

**DOI:** 10.4103/0972-2327.41878

**Published:** 2008

**Authors:** Sunil Raina, Sushil Razdan, Kamal K. Pandita, Sujeet Raina

**Affiliations:** Department of Community Medicine, Acharya Shree Chander College of Medical Sciences, Sidhra, Jammu-181 001 (J and K), India; 1 Department of Neurology, Acharya Shree Chander College of Medical Sciences, Sidhra, Jammu-181 001 (J and K), India; 2 Department of Medicine, Acharya Shree Chander College of Medical Sciences, Sidhra, Jammu-181 001 (J and K), India; 3 Department of Medicine, Indira Gandhi Medical College, Shimla - 171 001, HP, India

**Keywords:** Dementia, Kashmiri migrants, prevalence

## Abstract

**Background:**

Neurological diseases are common disorders resulting in the loss of productive life and disability. Dementia is becoming a major public health problem in the developing world also.

**Aim:**

To ascertain the prevalence of dementia among Kashmiri Pandit population aged 60 years and above.

**Materials and Methods:**

A cross-sectional survey was conducted among the elderly population of the Kashmiris living in a migrant camp. We developed and used a Kashmiri version of the Mini-Mental State Examination as the test instrument, and a score below 24 was considered indicative of dementia. A functional ability questionnaire was also administered to the subjects. A neurologist carried out the examinations.

**Results:**

A sample comprising 200 subjects (95 males and 105 females) were evaluated. The prevalence of dementia is 6.5% among the Kashmiri Pandit population aged 60 years and above, which is higher than that reported from other parts of India.

## Introduction

The prevalence of dementia is increasing with the increase in global life expectancy. It is a disease strongly associated with increasing age. Dementia is a major public health problem among the elderly in industrialized countries. It can also have a devastating impact on developing countries whose population is aging most rapidly. The most remarkable effects of population aging are expected in the most rapidly developing regions such as China, India and Latin America.[1] By the year 2020, 70% of the world population aged above 60 years will be that in developing countries, with 14.2% in India.[2] Elderly persons are at a high risk for disease and disability. This aging population will place urgent demands on the health care systems developing countries most of which are ill-prepared for fulfilling such demands. The World Health Organization (WHO) projects that by the year 2020, Africa, Asia, and Latin America will have more than 55 million people with senile dementia.[2]

This cross-sectional epidemiological study aimed to estimate the prevalence of dementia in the elderly Kashmiri migrant population in a community cluster at Mishriwala camp, Jammu.

## Materials and Methods

### Sample area and study population

Mishriwala camp is a community cluster of Kashmiri migrant population, and is located 12 km west of Jammu city in northern India. This camp was established in 1990 to accommodate those Kashmiri Pandit families who had left the Kashmir valley in the wake of militancy in Kashmir. Since its establishment 18 years ago, this community cluster has seen the lowest immigration and emigration.

The prevalence cohort consisted of 200 individuals aged 60 years and above residing in the Mishriwala migrant community cluster of Jammu city. Subjects were selected after conducting a house-to-house survey and verification of age from the ration cards. Their ages were further confirmed in person by reference to personnel and historical sentinel events as is the standard research practice in developing countries.[3] All participating subjects provided informed consent.

### Instrument development

Procedures for tool development were same as the standard followed by Chandra *et al.*[4] The most common language that the study population could understand was Kashmiri. The instrumentation appropriate for the local population was developed with the help of a neurologist, physicians and epidemiologist, all of whom were Kashmiri. The selected instrument was a Kashmiri version of Mini-Mental State Examination (MMSE) analogous to the standard MMSE. This was a systematic, iterative process. The team of clinicians (neurologist, internists and epidemiologist), all bilingual, i.e., Kashmiri as well as English speaking, selected the items in the MMSE instrument and translated them from English to Kashmiri, with a different group back translating the responses to English. The selected instrument was first pretested among educated, bilingual Kashmiri Pandit population of the Mishriwala migrant camp and then pilot-tested on a random sample of 50 purely Kashmiri-speaking migrant population in the Mishriwala camp. The Kashmiri version of MMSE was administered by trained interviewers to all 200 individuals of 60 years or above at their residences. Using the same iterative process, a functional ability scale, in the questionnaire form (Everyday Abilities Scale for India; EASI) for administration to the subject and a family member, was developed for this population. It included items related to older adults' routine activities in this rural Indian setting. The test items were examined and modified to maximize the ease of administration and comprehension, acceptability to study participants and reliability of test administration and scoring. This helped us to obtain the functional ability data in subjects who were cognitively untestable because of sensory impairment, illness or severe dementia. An MMSE score below 24 (out of a possible score of 30) was evaluated for clinical diagnosis. This scoring was performed to establish the presence or absence of a dementia syndrome, stage of severity and the likely cause. The MMSE has been used as a screening tool to detect dementia.[5,6]

Clinical evaluation was carried out by a neurologist with the help from two internists. The evaluation included the detailed history, general physical examination, neurological examination and the examination of the mental status of the subject. Help from an epidemiologist was received to corroborate the evidence by simultaneously conducting an interview. Patients diagnosed with dementia were subject to further detailed laboratory investigations such as hemogram, biochemistry screening, thyroid function tests and magnetic resonance imaging (MRI) scan.

## Results

In the sample comprising 200 elderly subjects, 95 (47.5%) were males and 105 (52.5%) females. The numbers of patients in the different age groups are shown in Table 1. Demographic characteristics and the prevalence of dementia in the study population are shown in Table 2. There is a predominantly illiterate study population comprising 100 (50%) individuals, including both males and females. The overall age-specific prevalence of dementia was 6.5%. The highest rates were observed in subjects in the age group of 65–69 years with the prevalence of 12.9%.The prevalence rate of dementia in males was 8.4% and that in females was 4.7%. The results of laboratory investigations performed in all 13 patients with dementia revealed normal hemogram, renal and liver functions. The thyroid profile was also normal in all the patients. The MRI of the brain could be performed in only four patients and all images obtained were normal.

**Table 1:** Age and gender distribution of the study population

Age (years)	Male	Female
60–64	30	50
65–69	30	32
70–74	22	18
≥75	13	05
Total	95	105

**Table 2:** Demographic characteristics and prevalence of dementia in the study population (N = 200)

Variable	Total number of subjects	No. of cases of dementia	Prevalence of dementia (%)
Age group			
60–64	80	2	2.5
65–69	62	8	12.9
70–74	40	2	5
≥75		18	15.5
Gender			
Male	95	8	8.4
Female	105	5	4.7
Literacy			
Illiterate	100	5	5
Primary			
education	32	6	18
Matriculation	46	2	4.3
Above	22	0	-

## Discussion

The number of people with dementia in the population at a given time point is considered as its prevalence. Throughout the world, a large number of surveys have been carried out to find the prevalence of dementia with variable results. All results show a marked increase in the prevalence with the increase in age. A statistical integration of results from a number of prevalence studies resulted the following figures.[7]

Data from developing and less developed countries is sparse, and it is difficult to estimate the world-wide prevalence of dementia. According to the 2001 census, India is home to more than 76 million people aged 60 years and above.[1] This age group, currently comprising only 7.4% of the population, is expected to grow dramatically in the next few decades. It is estimated that there are already approximately 1.5 million people affected by dementia in India, and this number is likely to increase by 300% in the next four decades.[8] Several community-based urban and rural studies on dementia from different parts of India have documented lower rates varying from 1.02% to 3.36% above 60–65 years of age.[9–15] These rates are comparatively less in comparison to a higher prevalence of dementia in developed countries, which ranges between 5–10% after 60–65 years of age.[16] Dementia appears to be very rare in the native Kashmiri population.[17] However, our study revealed dementia in a substantial population of the Kashmiri migrant population. Consistent with previous studies conducted on its prevalence in Asian and Western countries, the rate of dementia has been found to increase with the advancing age. The prevalence rate of dementia as estimated in our study is 6.5%, which is higher than that reported from other parts of India and in other Asian countries. Thus, the differences between the prevalence rate in our study and those of other studies in India may be a function of a valid regional difference. Adverse social outcomes such as social disengagement, stress associated with migration from native homes and hearths, differences in lifestyle, longer life expectancy, health awareness and healthcare delivery systems may be the factors contributing to this difference. The prevalence of dementia among illiterates was 5%; among subjects with primary level education, 18% and among subjects with matric level education, 4.3%.

In conclusion, we found that the prevalence of dementia is higher than that reported by other studies in India. The prevalence of the dementia syndrome increased with age. The future research in our prevalence cohort will be a follow-up study to estimate the incidence of dementia among Kashmiri migrant population.
